# Establishing associated risk factors, including fungal and parasitic infections among Malaysians living with schizophrenia

**DOI:** 10.1038/s41598-023-50299-7

**Published:** 2024-01-03

**Authors:** Freddy Franklin, Arutchelvan Rajamanikam, Wei Kit Phang, Chandramathi Samudi Raju, Jesjeet Singh Gill, Benedict Francis, Luke Sy-Cherng Woon, Suresh Kumar Govind

**Affiliations:** 1 Department of Parasitology, Universiti Malaya (UM), Kuala Lumpur, Malaysia; 2 Department of Medical Microbiology, Universiti Malaya (UM), Kuala Lumpur, Malaysia; 3 Department of Psychological Medicine, Faculty of Medicine, Universiti Malaya, Kuala Lumpur, Malaysia; 4 Department of Psychiatry, Faculty of Medicine, Universiti Kebangsaan Malaysia (UKM), Bangi, Malaysia

**Keywords:** Microbiology, Psychology

## Abstract

The aetiology of schizophrenia is multifactorial, and the identification of its risk factors are scarce and highly variable. A cross-sectional study was conducted to investigate the risk factors associated with schizophrenia among Malaysian sub-population. A total of 120 individuals diagnosed with schizophrenia (SZ) and 180 non-schizophrenic (NS) individuals participated in a questionnaire-based survey. Data of complete questionnaire responses obtained from 91 SZ and 120 NS participants were used in statistical analyses. Stool samples were obtained from the participants and screened for gut parasites and fungi using conventional polymerase chain reaction (PCR). The median age were 46 years (interquartile range (IQR) 37 to 60 years) and 35 years (IQR 24 to 47.75 years) for SZ and NS respectively. Multivariable binary logistic regression showed that the factors associated with increased risk of SZ were age, sex, unemployment, presence of other chronic ailment, smoking, and high dairy consumption per week. These factors, except sex, were positively associated with the severity of SZ. Breastfed at infancy as well as vitamin and supplement consumption showed a protective effect against SZ. After data clean-up, fungal or parasitic infections were found in 98% (39/42). of SZ participants and 6.1% (3/49) of NS participants. Our findings identified non-modifiable risk factors (age and sex) and modifiable lifestyle-related risk factors (unemployment, presence of other chronic ailment, smoking, and high dairy consumption per week) associated with SZ and implicate the need for medical attention in preventing fungal and parasitic infections in SZ.

## Introduction

The prevalence of mental disorders among Malaysian adults was reported to be approximately 29% in 2015 and the figure is estimated to increase in the future^1,2^. Mental illness imposes a heavy financial burden on the healthcare systems^3^ and a study reported that approximately half of Malaysians cannot afford mental health care. SZ is a neuropsychiatric disorder that affects 0.5–1% of the human population^4–6^ and the incidence rate of SZ in Malaysia has been reported to be around 0.04%^7^. The risk factors are defined as something that predispose or contribute to the development of an illness. The risk factors of SZ are multifactorial, which include genetics and environmental factor leading to brain anomaly^8,9^. Currently, there is a scarcity of studies investigating the association of commonly studied risk factors such as sociodemographic factors^10^, dietary habits^11,12^, and lifestyle choices^13,14^ especially among Malaysian SZ population.

In recent times, attention has shifted to assessing the influence of gut microbiota in the pathogenesis of mental illness. Trillions of microorganisms, including bacteria, parasites, and fungi, live in the human intestine and make up the gut microbiota^15^. Parasitic infections such as *Toxoplasma gondii* have also been established as a risk factor for SZ^16,17^. Recently, we reported a prevalence of 40% of *Blastocystis* sp. infection among chronic SZ patients^18^. Another study reported a 55% prevalence of *Blastocystis* sp*.* infection among hospitalized male SZ patients in Iran^19^. On the same note, trends of certain fungal^20–22^ and parasitic abundances^23–26^ in the gut have been reported among individuals with different mental illnesses and mood disorders as well. Intestinal parasites and fungi have been reported to alter the composition of the gut microbiome^27–29^ and via gut-brain axis, altering the brain chemistry^30,31^. Intestinal dysbiosis can trigger inflammatory pathway which may contribute worsening the symptoms of autoimmune and neuropsychiatric disorders^32^. Therefore intestinal parasitic and fungal infection among individuals with mental illness may exacerbates existing symptoms. As of today, intestinal parasitic and fungal infection as a potential risk factor in SZ population in Malaysia is still unknown.

In this study, fungal and parasitic infections as a risk factor in SZ patients in Selangor, Malaysia were assessed. To our knowledge, this is the first study in Malaysia that highlights the risk factors of schizophrenia and presented the association between SZ and gut parasitic/fungal infections.

## Results

**Figure 1:**
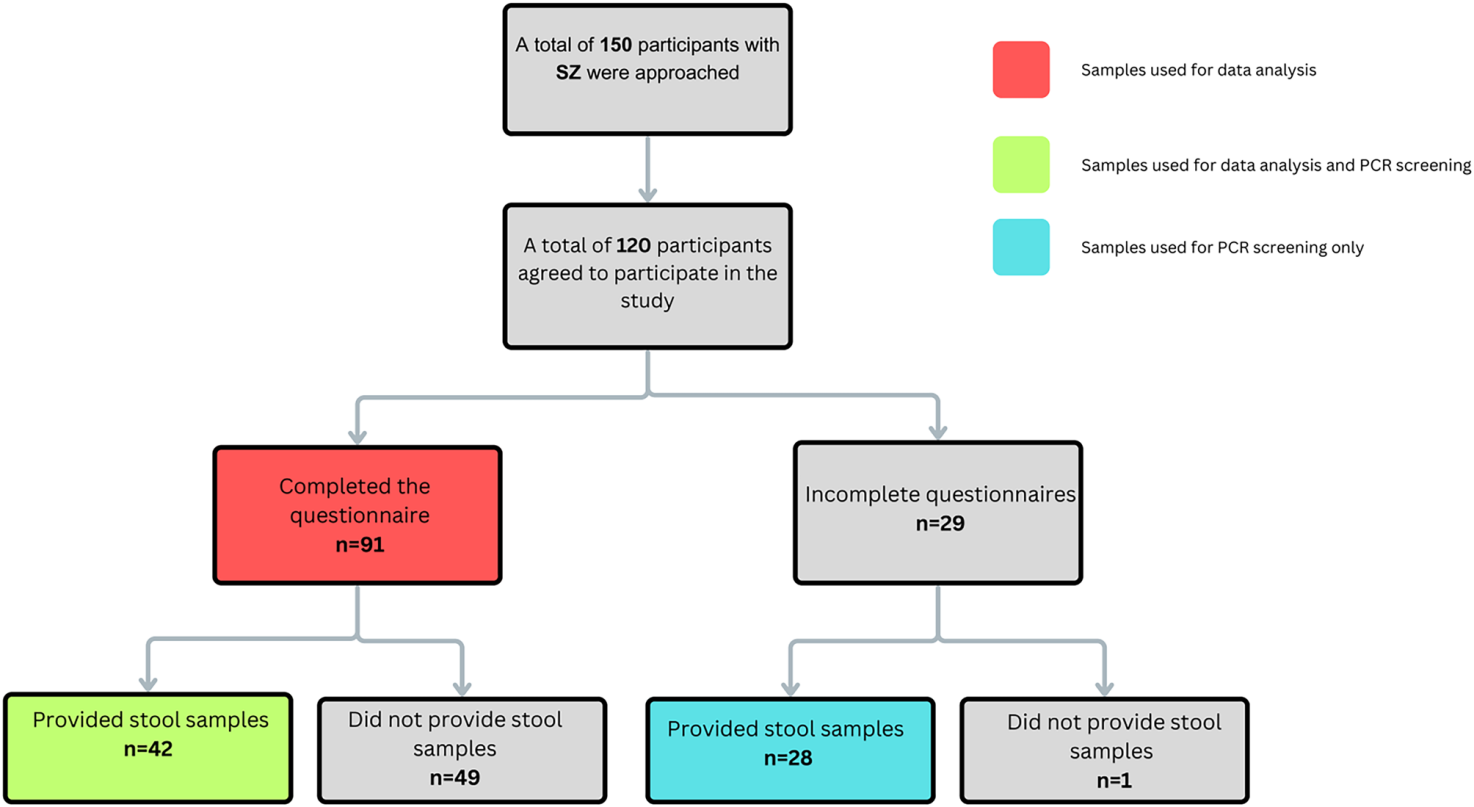
Flow chart for recruitment of patients for the administration of questionnaire and stool collection for fungal and parasitic screening in individuals with Schizophrenia (SZ).

**Figure 2:**
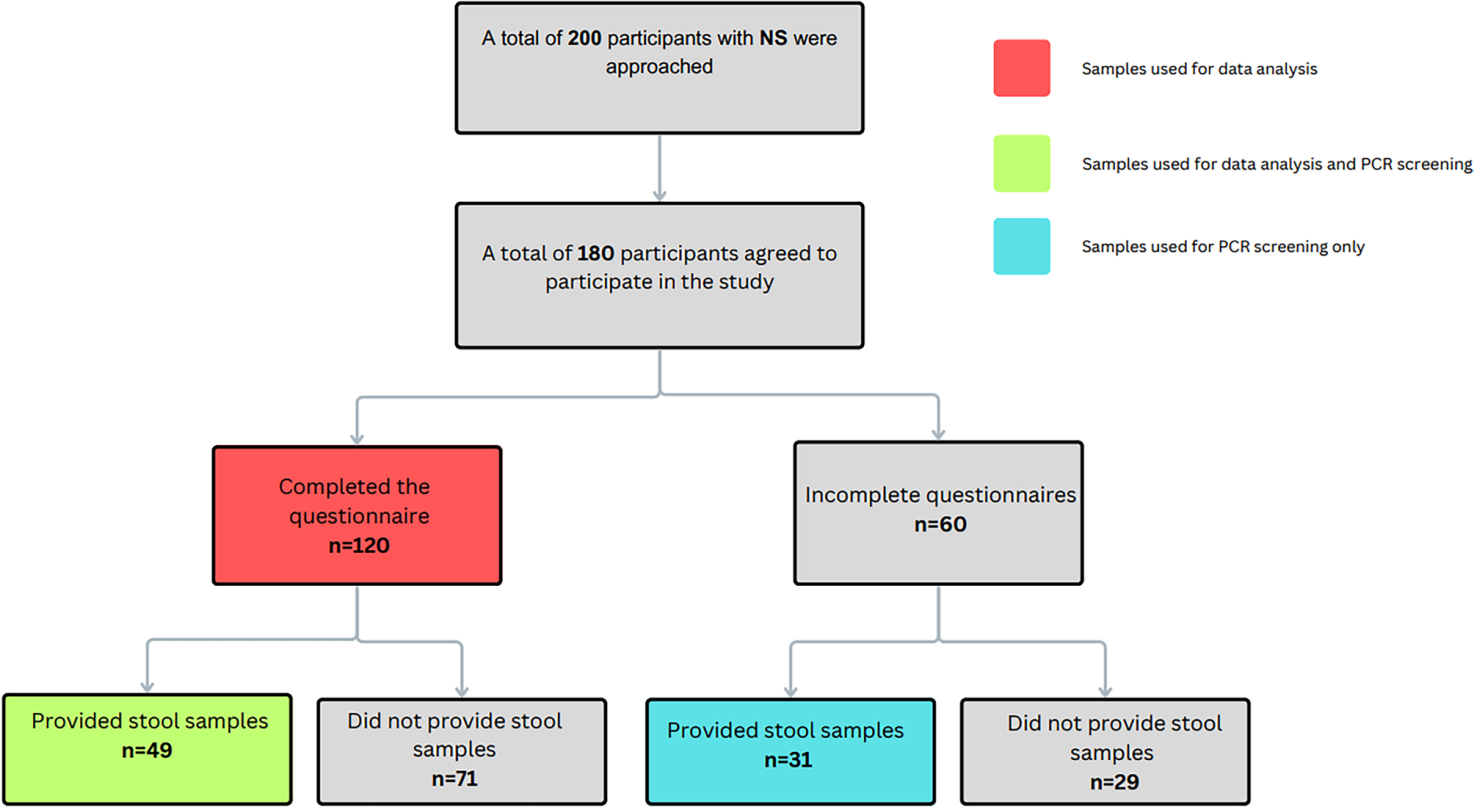
Flow chart for recruitment of patients for the administration of questionnaire and stool collection for fungal and parasitic screening in individuals without Schizophrenia (NS).

A total of 120 SZ participants agreed to participate in the study. After filtering out incomplete questionnaire, the total of SZ participants were brought to 91 participants for data analysis. Out of the 120 participants with SZ, a total of 70 patients provided stool samples (42 participants with complete questionnaire and 28 with incomplete questionnaires). While all 70 samples were subjected to fungal and parasitic screening for prevalence, only 42 samples with the completed questionnaires were used for data analysis. A flow chart on the sample size of SZ is depicted in Fig. 1. On the other hand, a total of 180 NS participants agreed to participate in the study (Fig. 2). After filtering the incomplete questionnaires, the sample size was brought to 120 individuals for data analysis. A total of 80 individuals from the NS cohort provided stool samples (49 individuals with complete questionnaires and 31 individuals with incomplete questionnaires).

Data analysis was carried out in three sections. Section A was to identify significant risk factors among the SZ population through univariable and multivariable analysis. In section B, we analysed the association of the aforementioned risk factors to the CGI-S score (severity) while in Section C the risk of the prevalence of fungal and/or parasitic infection among the SZ cohort group was assessed.

## Section A: Analysis of risk factors among the SZ population

In Section A, we assessed the sociodemographic of SZ and NS individuals and conducted univariable and multivariable binary logistic regression to identify risks that associates with SZ. A total of 211 participants [patients with schizophrenia 91 (43.1%) and 120 (56.9%) individuals without schizophrenia] participated in this study (Tables 1 and 2). The median age of SZ was 46.0 (IQR: 37 to 60) ± 15.1, while NS had a median age of 35.0 (IQR:24 to 47.75). There was a significant difference (p < 0.001) in the median age associated with aging as a potential risk factor for SZ (Table 3). Several factors including ethnicity, mode of delivery, family history of mental illness, dairy consumption, lower intake of vegetables and fruits and higher white meat intakes were found to be associated with SZ in univariable binary logistic regression (Table 3). However, some of these factors were not statistically significant in the subsequent multivariable binary logistic regression (Table 4).

**Table 1:** Sociodemographic characteristics of participants in this study (n = 211).

Variables	SZ (n = 91)		NS (n = 120)		Total	
	Median	IQR	Median	IQR		
Age (year)*	46	(37 to 60)	35	(24 to 47.75)		
Height (cm)*	165	(157 to 173)	165	(157.25 to 169.75)		
Weight (kg)*	65	(56 to 80)	64.5	(55 to 74)		

	n	%	n	%	n	%
Sex						
Male	60	52.17	55	47.83	115	100.00
Female	31	32.29	65	67.71	96	100.00
Ethnicity						
Malay	19	31.15	42	68.85	61	100.00
Chinese	51	53.68	44	46.32	95	100.00
Indian	21	38.18	34	61.82	55	100.00
Occupation						
Employed	23	28.40	58	71.60	81	100.00
Unemployed	68	52.31	62	47.69	130	100.00
Mode of delivery						
Vaginal	81	44.75	100	55.25	181	100.00
Caesarean	10	33.33	20	66.67	30	100.00
Location of delivery						
Hospital	71	40.34	105	59.66	176	100.00
Home	20	57.14	15	42.86	35	100.00
Breastfed during infancy						
No	36	64.29	20	35.71	56	100.00
Yes	55	35.48	100	64.52	155	100.00
Family history of mental illness						
No	65	38.24	105	61.76	170	100.00
Yes	26	63.41	15	36.59	41	100.00
Presence of other ailments^a^						
No	68	39.08	106	60.92	174	100.00
Yes	23	62.16	14	37.84	37	100.00
Presence of gastrointestinal symptoms						
No	68	42.24	93	57.76	161	100.00
Yes	23	46.00	27	54.00	50	100.00
Severity of SZ (CGI-S score)						
Normal	0	0.00	85	100.00	85	100.00
Mild	26	57.78	19	42.22	45	100.00
Moderate	18	72.00	7	28.00	25	100.00
Severe	47	83.93	9	16.07	56	100.00

*Age, height, and weight are expressed in median (IQR).

^a^Presence of any other chronic ailments such as hypertension, renal failure, diabetes etc.

**Table 2:** Lifestyle and dietary habits of participants in this study (N = 211).

Variables	SZ		NZ		Total	
	n	%	n	%	n	%
Frequency of vigorous workout (per week)						
≤ 3	59	42.75	79	57.25	138	100.00
> 3	32	43.84	41	56.16	73	100.00
Frequency of moderate workout (per week)						
≤ 3	49	45.79	58	54.21	107	100.00
> 3	42	40.38	62	59.62	104	100.00
Frequency of destress (per week)						
≤ 3	62	45.59	74	54.41	136	100.00
> 3	29	38.67	46	61.33	75	100.00
Smoking^a^						
No	62	38.51	99	61.49	161	100.00
Yes	29	58.00	21	42.00	50	100.00
Consumption of alcohol^b^						
No	81	48.80	85	51.20	166	100.00
Yes	10	22.22	35	77.78	45	100.00
Type of nutrition						
Omnivorous	87	43.72	112	56.28	199	100.00
Non-omnivorous	4	33.33	8	66.67	12	100.00
Consumption of probiotics						
No	57	44.88	70	55.12	127	100.00
Yes	34	40.48	50	59.52	84	100.00
Consumption of vitamins and supplement						
No	76	46.91	86	53.09	162	100.00
Yes	15	30.61	34	69.39	49	100.00
Consumption of fruits (portions per week)						
0 to 1	29	37.66	48	62.34	77	100.00
2 to 3	26	53.06	23	46.94	49	100.00
4 to 5	10	41.67	14	58.33	24	100.00
> 5	26	42.62	35	57.38	61	100.00
Consumption of vegetables (portions per week)						
0 to 1	19	47.50	21	52.50	40	100.00
2 to 3	18	33.96	35	66.04	53	100.00
4 to 5	12	37.50	20	62.50	32	100.00
> 5	42	48.84	44	51.16	86	100.00
Consumption of dairy (portions per week)						
0 to 1	26	33.33	52	66.67	78	100.00
2 to 3	12	29.27	29	70.73	41	100.00
4 to 5	16	53.33	14	46.67	30	100.00
> 5	37	59.68	25	40.32	62	100.00
Consumption of fish (portions per week)						
0 to 1	46	42.20	63	57.80	109	100.00
2 to 3	22	44.00	28	56.00	50	100.00
4 to 5	12	48.00	13	52.00	25	100.00
> 5	11	40.74	16	59.26	27	100.00
Consumption of red meat (portions per week)						
0 to 1	66	42.86	88	57.14	154	100.00
2 to 3	12	40.00	18	60.00	30	100.00
4 to 5	2	28.57	5	71.43	7	100.00
> 5	11	55.00	9	45.00	20	100.00
Consumption of red meat (portions per week)						
0 to 1	17	32.08	36	67.92	53	100.00
2 to 3	9	34.62	17	65.38	26	100.00
4 to 5	16	41.03	23	58.97	39	100.00
> 5	49	52.69	44	47.31	93	100.00

^a^Smokes at least once per week.

^b^Consumes alcohol at least once per week.

**Table 3:** Univariable binary logistic regression analysis of risk factors associated with schizophrenia.

Variables	COR (95% CI)	p-value
Age (year)	1.06 (1.03–1.08)	< 0.001*
Height (cm)	1.01 (0.99–1.03)	0.308
Weight (kg)	1.01 (0.99–1.02)	0.479
Sex		
Male	2.29 (1.30–4.02)	0.004*
Female	1	
Ethnicity		
Malay	1	
Chinese	2.56 (1.30–5.04)	0.006*
Indian	1.37 (0.63–2.94)	0.427
Occupation		
Employed	1	
Unemployed	2.77 (1.53–5.01)	0.001*
Mode of delivery		
Vaginal	1.62 (0.72–3.67)	0.245*
Caesarean	1	
Location of delivery		
Hospital	1	
Home	1.97 (0.95–4.11)	0.070*
Breastfed during infancy		
No	1	
Yes	0.31 (0.16–0.58)	< 0.001*
Family history of mental illness		
No	1	
Yes	2.80 (1.38–5.68)	0.004*
Presence of other ailments^a^		
No	1	
Yes	2.56 (1.23–5.32)	0.012*
Presence of gastrointestinal symptoms		
No	1	
Yes	1.17 (0.62–2.21)	0.639
Frequency of vigorous workout (per week)		
≤ 3	1	
> 3	1.05 (0.59–1.85)	0.880
Frequency of moderate workout (per week)		
≤ 3	1	
> 3	0.80 (0.47–1.38)	0.428
Frequency of destress (per week)		
≤ 3	1	
> 3	0.75 (0.42–1.33)	0.332
Smoking^b^		
No	1	
Yes	2.21 (1.16–4.2)	0.016*
Consumption of alcohol^c^		
No	1	
Yes	0.30 (0.14–0.65)	0.002*
Type of nutrition		
Omnivorous	1	
Non-omnivorous	0.64 (0.19–2.21)	0.484
Consumption of probiotics		
No	1	
Yes	0.84 (0.48–1.46)	0.527
Consumption of vitamins and supplement		
No	1	
Yes	0.46 (0.25–0.99)	0.046*
Consumption of fruits (portions per week)		
0 to 1	1	
2 to 3	1.87 (0.91–3.87)	0.091*
4 to 5	1.18 (0.47–3.01)	0.725
> 5	1.23 (0.62–2.44)	0.555
Consumption of vegetables (portions per week)		
0 to 1	1	
2 to 3	0.57 (0.25–1.32)	0.188*
4 to 5	0.66 (0.26–1.71)	0.395
> 5	1.06 (0.50–2.24)	0.889
Consumption of dairy (portions per week)		
0 to 1	1	
2 to 3	0.57 (0.36–1.88)	0.651
4 to 5	0.66 (0.97–5.39)	0.059*
> 5	1.06 (1.48–5.91)	0.002*
Consumption of fish (portions per week)		
0 to 1	1	
2 to 3	1.08 (0.55–2.12)	0.832
4 to 5	1.26 (0.53–3.02)	0.598
> 5	0.94 (0.40–2.22)	0.890
Consumption of red meat (portions per week)		
0 to 1	1	
2 to 3	0.89 (0.40–1.97)	0.772
4 to 5	0.53 (0.10–2.84)	0.461
> 5	1.63 (0.64–4.16)	0.307
Consumption of white meat (portions per week)		
0 to 1	1	
2 to 3	1.12 (0.42–3.03)	0.821
4 to 5	1.47 (0.62–3.48)	0.377
> 5	2.36 (1.16–4.78)	0.017*

*COR* crude odds ratio, *CI* confidence interval.

*Statistical significance (p-value < 0.25).

^a^Presence of any other chronic ailments such as hypertension, renal failure, diabetes etc.

^b^Smokes at least once per week.

^c^Consumes alcohol at least once per week.

**Table 4:** Multivariable binary logistic regression analysis of risk factors associated with schizophrenia. All selected variables fulfill multicollinearity criteria. Following backward stepwise elimination, only variables with p < 0.05 were retained in the final model. Hosmer–Lemeshow test (*p* = 0.231) implies that the regression model is of good fit.

Variables	AOR (95% CI)	p-value
Age (year)	1.07 (1.04–1.11)	< 0.001*
Sex		
Male	3.69 (1.65–8.24)	< 0.001*
Female	1	
Occupation		
Employed	1	
Unemployed	4.65 (2.05–10.55)	< 0.001*
Breastfed during infancy		
No	1	
Yes	0.41 (0.17–0.95)	0.038*
Presence of other ailments^a^		
No	1	
Yes	4.03 (1.40–11.57)	0.010*
Smoking^b^		
No	1	
Yes	3.23 (1.30–8.06)	0.012*
Consumption of alcohol^c^		
No	1	
Yes	0.30 (0.11–0.81)	0.017*
Consumption of vitamins and supplement		
No	1	
Yes	0.37 (0.15–0.91)	0.031*
Consumption of dairy (portions per week)		
0 to 1	1	
2 to 3	0.49 (0.17–1.41)	0.183
4 to 5	2.37 (0.76–7.37)	0.137
> 5	3.08 (1.27–7.46)	0.002*

*AOR* adjusted odds ratio, *CI* confidence interval.

*Statistical significance (p < 0.05).

^a^Presence of any other chronic ailments such as hypertension, renal failure, diabetes etc. ^b^Smokes at least once per week. ^c^Consumes alcohol at least once per week.

The multivariable binary logistic regression analysis demonstrated that breastfed during infancy (adjusted odds ratio (AOR) 0.41; 95% confidence interval (CI): 0.17,0.95; p = 0.038) and consumption of vitamins and supplement (AOR 0.37; 95% CI:0.15,0.91; p = 0.031) as modifiable protective factors against SZ. Also, the risk of SZ was lower in participants who consume alcohol (AOR 0.30; 95% CI:0.11,0.81; p = 0.017). Nevertheless, the identified non-modifiable risk factors of SZ are advancing age (AOR 1.07; 95% CI:1.04,1.11; p < 0.001), and male sex (AOR 3.69; 95% CI:1.65,8.24; p < 0.001), whereas modifiable risk factors of SZ are unemployment (AOR 4.65; 95% CI:2.05,10.55; p < 0.001), smoking (AOR 3.23; 95% CI:1.30,8.06; p = 0.012), presence of other ailments (AOR 4.03; 95% CI:1.40,11.57; p = 0.011), and high (> 5 portions per week) consumption of dairy products (AOR 3.08; 95% CI:1.27,7.46; p = 0.002).

## Section B: Analysis of risk factors of Schizophrenia based on the severity of the symptoms via CGI-S Score

In Section B, several factors were found positively associated with severity score of CGI-S through ordinal logistic regression. The risk factors that were significantly associated with severity of SZ include age (AOR 1.06; 95% CI 1.04, 1.09; p < 0.001), unemployment (AOR 4.54; 95% CI 2.34, 8.81; p < 0.001), presence of other ailments (AOR 2.44; 95% CI 1.09, 5.45; p = 0.006), smoking (AOR 2.50; 95% CI 1.27, 4.94; p = 0.001), and high dairy consumption (AOR 2.82; 95% CI 1.37, 5.81; p = 0.005) (Table 5).

**Table 5:** Multivariable ordinal logistic regression analysis of risk factors associated with schizophrenia based on CGI-S.

Variables	AOR (95% CI)	p-value	Brant test p-value
Age (year)	1.06 (1.04–1.09)	< 0.001*	0.881
Occupation			
Employed	1		
Unemployed	4.54 (2.34–8.81)	< 0.001*	0.678
Presence of other ailments^a^			
No	1		
Yes	2.44 (1.09–5.45)	0.029*	0.223
Smoking^b^			
No	1		
Yes	2.50 (1.27–4.94)	0.008*	0.695
Consumption of vitamins and supplement			
No	1		
Yes	0.38 (0.18–0.82)	0.013*	0.088
Consumption of dairy (portions per week)			
0 to 1	1		
2 to 3	0.61 (0.25–1.50)	0.280	0.366
4 to 5	1.55 (0.63–3.83)	0.343	0.749
> 5	2.82 (1.37–5.81)	0.005*	0.613

Brant test with p-value ≥ 0.05 indicates that the respective variable fulfills the proportional odds assumption of the model. Ordinal Hosmer–Lemeshow test (p = 0.639) implies that the regression model is of good fit.

*AOR* adjusted odds ratio, *CI* confidence interval.

*Statistical significance (p < 0.05).

^a^Presence of any other chronic ailments such as hypertension, renal failure, diabetes etc.

^b^Smokes at least once per week.

## Section C: Prevalence of fungal and parasitic infections among people with schizophrenia

In Section C, we analysed the significance of fungal and parasitic infection (*Blastocystis* sp., *Cryptosporidium* sp., Microsporidium (*Enterocytozoon bieneusi*), *Entamoeba* sp., *Candida* sp. and *Aspergillus* sp.) among 91 individuals that provided stool samples and with completed questionnaires (42 individuals with SZ and 49 individuals from NS cohort). Overall, the prevalence of fungal or parasitic infection among SZ and NS group was 64% (45/70) and 6.25% (5/80) respectively. However, after the filtration of data in accordance to completed questionnaires, the prevalence of fungal and parasitic infections among SZ and NS group was brought to 98% (39/42) and 6.1% (3/49) respectively.

Majority of the fungal and parasitic infection (SZ: 50/52 and NS: 3/3) were successfully screened using conventional PCR when compared with microscopy (Supplementary Table S2 and S3). Most common group were *Candida* and *Microsporidium*, which were detected in 15 samples each (21%) via conventional PCR. In addition, 9 *Blastocystis* sp. positive samples (13%) were of detected via both microscopy and PCR. Formalin ether sedimentation did not detect any samples positive for *Blastocystis* sp. cysts. On the other hand, only six out the eight *Cryptosporidium* sp. were detected via PCR with the three being detected through modified ZN staining method.

**Figure 3:**
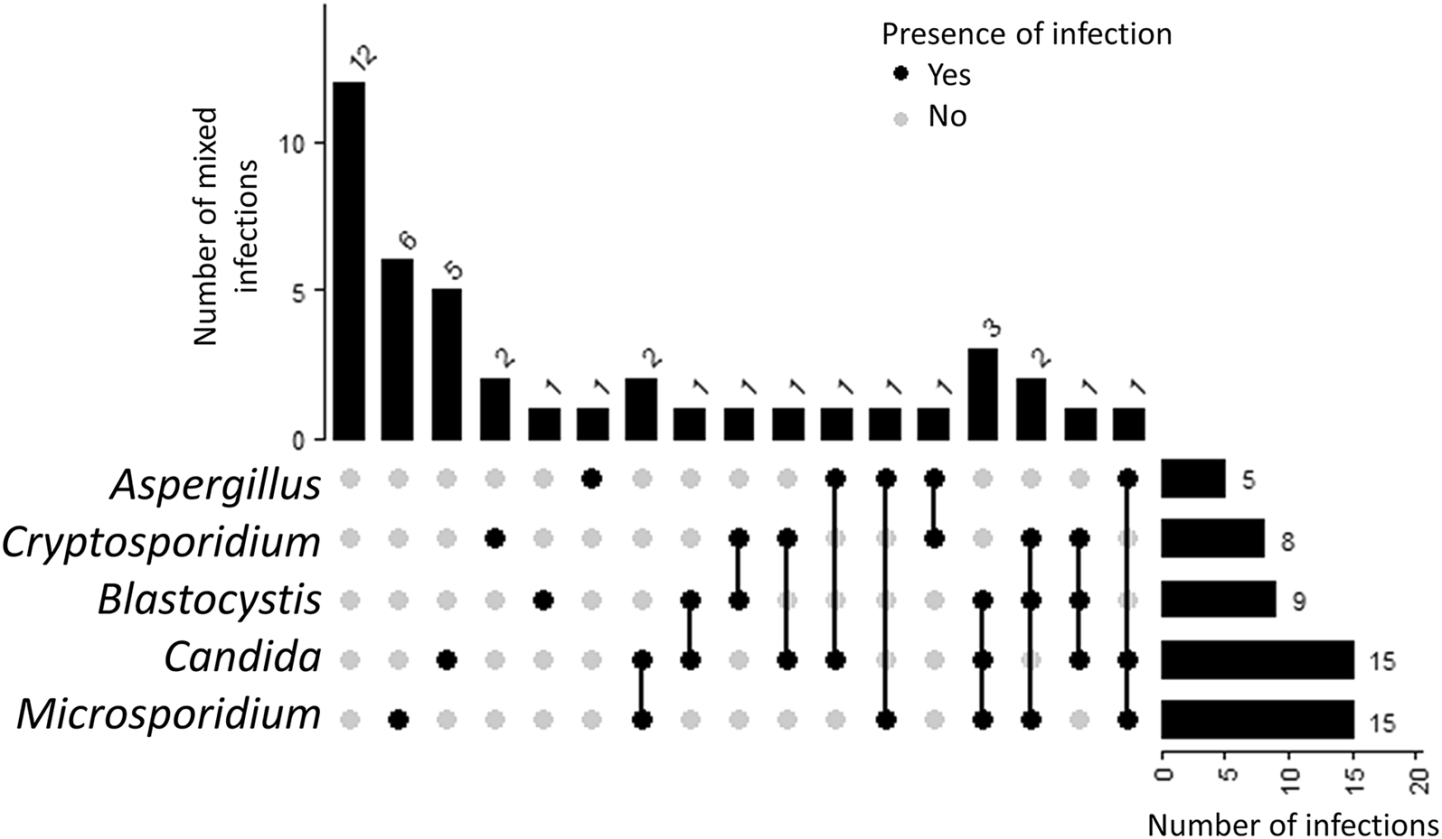
UpSet diagram illustrates the number of mixed infections in SZ patients (n = 42). The bar graph to the right indicate the number of infections screened in patient stool samples by pathogen group. The connected nodes indicate the concurrent infection by different pathogen group.

**Figure 4:**
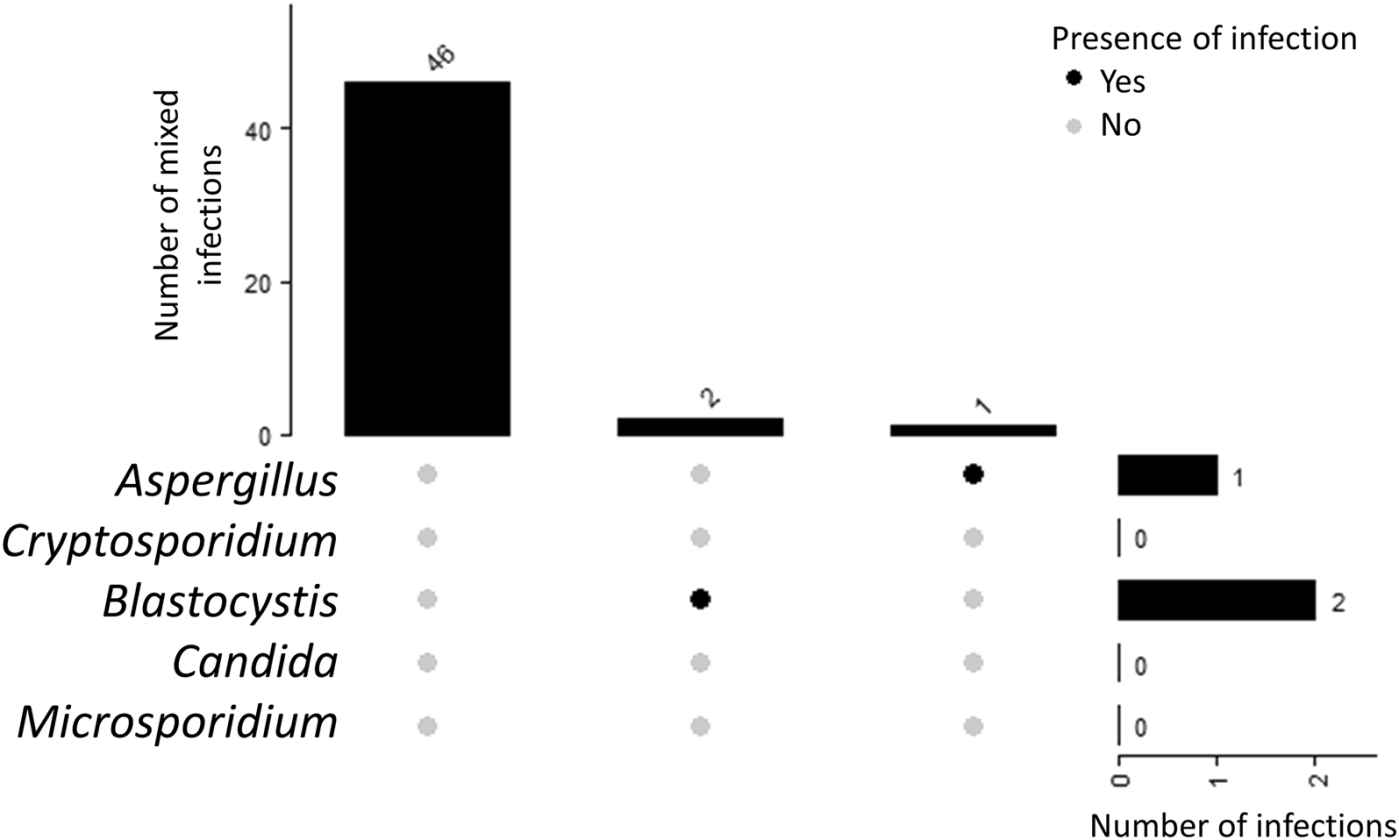
UpSet diagram illustrates the number of infections in NS patient stool samples (n = 49). The bar graph to the right indicate the number of infections screened in patient stool samples by pathogen group.

**Figure 5:**
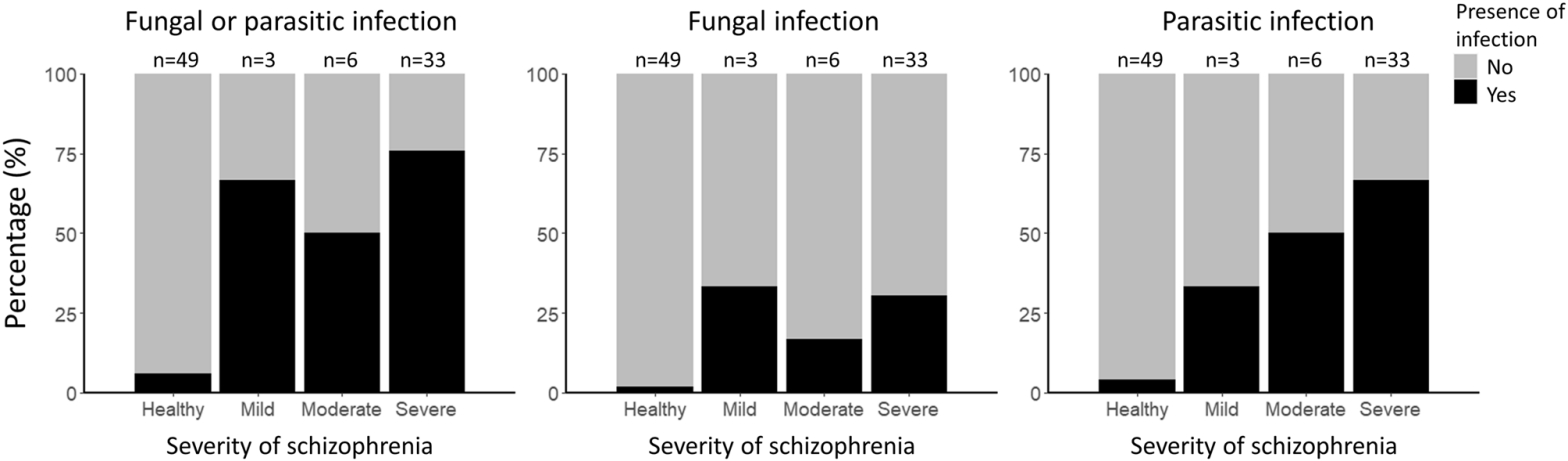
Percentage of fungal or parasitic infection by severity of schizophrenia. The figure shows the prevalence of fungal or parasitic infection among SZ and NS cohort group after the filtration of questionnaires. All NS individuals are categorized as the healthy group whereas SZ individuals are categorized into mild, moderate, and severe groups.

Fifteen (n = 15; 35.71%) individuals with SZ were detected with mono-infection of either fungal or parasite (Fig. 3). In contrast, double-infections and triple-infections were detected in 8 (19.05%) and 7 (16.67%) patient samples, respectively. A total of 12 individuals with SZ did not have any infection. In contrast, majority of the NS samples did not have any fungal or parasitic infections (n = 46; 93.88%) (Fig. 4) with some individuals having mono-infections of *Blastocystis* sp. (n = 2; 4.08%) and *Aspergillus* sp. (n = 2; 2.04%) were detected. We found significant association between fungal and parasitic infection in the SZ cohort group (p = 0.003), with majority of the SZ individuals having an either fungal or parasitic infection (Tables 6 and 7). Majority of the fungal and parasitic infection was observed among the SZ individuals with severe CGI-S (Fig. 5).

**Table 6:** Multivariable binary logistic regression analysis indicates association between fungal or parasitic infection and schizophrenia.

Variables	AOR (95% CI)	p-value
Age (year)	1.24 (1.08–1.42)	0.003*
Consumption of vitamins and supplement		
No	1	
Yes	0.01 (0.0003–0.34)	0.011*
Consumption of dairy (portions per week)		
0 to 1	1	
2 to 3	1.63 (0.06–47.35)	0.775
4 to 5	11.59 (0.76–177.54)	0.079
> 5	113.53 (5.42–2380.35)	0.002*
Fungal or parasitic infection		
No	1	
Yes	281.06 (6.93–11,398.66)	0.003*

Hosmer–Lemeshow test (p = 0.462) implies that the regression model is of good fit.

**Table 7:** Multivariable ordinal logistic regression analysis of risk factors (fungal or parasitic infection) associated with schizophrenia based on CGI-S.

Variables	AOR (95% CI)	p-value	Brant test p-value
Age (year)	1.08 (1.03–1.12)	< 0.001*	0.268
Occupation			
Employed	1		
Unemployed	3.29 (1.02–10.60)	0.046*	0.891
Smoking^a^			
No	1		
Yes	4.44 (1.00–19.77)	0.050*	0.359
Fungal or parasitic infection			
No	1		
Yes	12.49 (3.84–40.62)	< 0.001*	0.497

Brant test with p-value ≥ 0.05 indicates that the respective variable fulfills the proportional odds assumption of the model. Ordinal Hosmer–Lemeshow test (p = 0.208) implies that the regression model is of good fit.

*COR* crude odds ratio, *CI* confidence interval.

*Statistical significance (p-value < 0.25).

^a^Smokes at least once per week.

## Discussion

The present study is the first to examine the associated risk factors of SZ among the urban Malaysian subpopulation**.** Our study revealed that older age, unemployment, not breastfed in infancy, vitamin and supplement consumption, smoking, high dairy consumption, and fungal or/and parasitic infection were significantly associated with SZ.

In our study population, most respondents had late(adult) onset of SZ and had at least one family member with SZ. The odds of developing SZ increases by 7% for each additionally yearly increase in age. While no concrete evidence has associated aging with the increased risk of having SZ, hypotheses have been made regarding the potential decline of cognitive health from aging as a possible contributor to SZ^33,34^. In addition, studies have reported SZ as a polygenic illness^9,35^, where genetics is one of the many possible aetiology of SZ. A recent study demonstrated genetic predisposition to mental health distinctly related to a specific brain structure and function that can be inherited^36^. Nevertheless, the multivariable analysis in this study did not find family history of SZ as an independent risk factor despite it was initially implicated as a significant factor in univariable analysis. Therefore, more populations specific studies with genome-wide association studies should shed light on the validity of this observation.

A significant percentage (n = 68,74%) of unemployment was observed among the SZ cohort. The odds of being unemployed were also seen to be increased with increasing severity of schizophrenia (AOR 4.54, 95% CI: 2.34,8.81). According to the Malaysian Employers Federation, the statutory retirement age is 60. Although the median age of SZ individuals were 46, unemployment was high in this group. A global study indicated the employment rate among SZ individuals to be around 40–60%^37^ and has been steadily declining for the last 50 years^38^. Stigmatization, discrimination, and the lack of access to mental healthcare have been cited as some of the factors for the low employment rate^39^. It has been reported that SZ individuals cannot sustain a job due to workplace discrimination and negative perceptions of their employability, which include being considered less intelligent, lacking in self-control, and being violent^40,41^. To the best of our knowledge, there are no data addressing employability issues among schizophrenic individuals from Malaysia have been reported. A recent study in Malaysia highlighted that the laws protecting employees with mental disabilities are severely lagging compared to other first-world countries^42^. However, observations from other countries have demonstrated that employment improves the cognitive function among SZ individual, particularly in attention and working memory^43,44^. Another study from Malaysian SZ population was in agreement with associations between cognitive functions and employment^45^. Thus, the present finding, not only highlight low employment in SZ population, it also raises concerns on the potential employability among individuals suffering from SZ especially in individuals with severe SZ.

Breastfeeding increases performance in cognitive tests and is negatively associated with various mental health disorders^46–48^. In parallel, our results demonstrated similar outcome where not being breastfed in infancy was a significant predictor of schizophrenic conditions. We noticed that, the odds of having SZ is reduced by 59% in breastfed individuals. There are various studies that have reported on the protective impacts of breastfed milk on SZ and potentially delayed onset of SZ symptoms^49–51^. Other studies have also reported that breastfeeding lowers the odds of developing other mental illnesses such as depression^28^ and body dysmorphia^52^. A recent study from Brazil suggested that longer breastfeeding duration during infancy had lower odds of developing severe mental disorders^53^.Our study was, however, restricted to only whether the participant was breastfed in their infancy without the consideration of duration. Unlike cow’s milk or most formula feeds, breast milk contains large amounts of long-chain polyunsaturated fatty acids, especially docosahexaenoic acid (DHA), an essential constituent of cell membranes in the central nervous system. Our observation concurs with previous findings that positively correlate breastfeeding with larger grey matter volume and thus potentially contribute towards better mental health and cognitive function^54,55^.

Consumption of vitamins and supplement were significantly associated to SZ in the studied population. The multivariate binary logistic regression implies that the occurrence of SZ was decreased by 63% in individuals consuming vitamins and supplements regularly. Although the duration of consumption and the type of supplement were not taken into consideration, our findings were in agreement with several other studies^56,57^ where consumption of certain vitamins such as vitamin B12 and folate is reported to improve the negative symptoms of schizophrenia^58^. Studies have indicated that SZ individuals, who are more prone to have dietary-related inflammation also had lower dietary pattern score^59^. Our findings demonstrated that consumption of dairy—more than 5 portions per week, was found to be a significant predictor of SZ. Dietary habits have been associated with being potential risk factors of SZ^60,61^ Nutrients, such as L-tryptophan, are important precursors for serotonin production whereas B12 and omega fatty acids are important for brain health and collectively they exert a positive effect on patients with mental illness^62–64^. One study has reported that high consumption of dairy products have been associated with poor outcome of SZ^65^. In contrast, high consumption of low-fat dairy has been reported to reduce depressive symptoms^66–68^.

Smoking and alcohol consumptions are often associated for SZ^69,70^. Studies have reported that the smoking rates among SZ are extremely high compared to healthy cohorts^32,42^. Our findings also suggest that smoking is significantly associated with higher CGI-S implicating its exacerbating effect on the severity of SZ and other mental illness^71,72^. In our observations, there was an elevated level of nicotine smoking among the SZ participants recruited where a total of 32% (29/32) of SZ were smokers. This percentage is higher when compared to the percentage of smokers among the Malaysian population (21%)^73^. Reportedly, SZ individuals consider smoking as a form of self-medication that helps alleviate the symptoms^74^. Most recently, studies have reported a molecular association between smoking and polygenic differences among SZ individuals who smoke^75,76^. A genome-wide association studies (GWAS) reported that the phylogenetic risk scores of SZ was significantly associated with long-term smoking^77,78^. It is said smoking contributes to neuronal abnormalities and alteration of gene expression that are responsible for neuronal development and can potentially impact the severity of SZ. The findings from the present study necessitates monitoring and control of nicotine smoking especially among SZ individual to manage the severity.

One fifth of people with SZ have alcohol use disorder^79^ and alcohol abuse have been reported to contribute psychosis^80–82^. In contrast, our study found a lower risk of SZ with alcohol consumption. However, this finding requires careful interpretation, as a significant portion of our SZ cohort did not consume alcohol. This could be because the participants were already on medication or resided in caring homes with strict prohibition against alcohol consumption. In addition, it should also be noted that National Health and Morbidity Survey (NHMS) carried out in the year 2019 reported that only 11.8% of Malaysians have consumed alcohol in the past 12 months^73^.

Remarkably, our findings demonstrated that more than half of the SZ individuals had fungal and parasitic infections as compared to the NS group. The significant association between infection and SZ implies that individuals with SZ is more likely to have fungal and parasitic infections or vice versa. Host and parasite interactions have been reported to trigger mental illness^83^. Toxoplasmosis^84^ and candidiasis^85^ have been reported as potential risk factors of SZ. In contrast, parasitic infections such as helminths have been used as a therapy through the gut brain axis for individuals with autism^86^. While it is apparent that gut parasite and fungus are key players that affect positively or negatively the cognitive and brain chemistry, more studies investigating the direct link of parasitic and fungal infection are needed to elucidate greater details of the gut brain linkage.

The gut microbiota is increasingly incriminated as a factor influencing the immunopathogenesis of SZ^87–89^. Intestinal dysbiosis due to multiple factors such as dietary and lifestyle habits can affect mental health through the gut-brain axis and gut inflammation^89,90^. Interestingly, risk factors of SZ seen in this study have been reported to impact the composition of the gut microbiota. Studies have reported that breastfeeding^91,92^, smoking^93,94^, dairy consumption^95,96^, and fungal and parasitic infections alters the gut microbiota and could potentially contribute to SZ via the already established gut-brain-axis. However, more studies need to be carried out to corroborate correlation and causality of gut microbiome and risk factors to the severity of SZ. Healthy lifestyle intervention is essential for individuals with SZ and as well towards populations at risk to reduce the possibility of getting SZ or increasing the severity of SZ for the existing SZ patients.

One of the limitations of our study was that the sampling carried out might not be reflective the entire Malaysian population as it was only carried out in two hospitals in Selangor Malaysia. However, it could be highly reflective of the urban population. Moreover, as a self-reporting questionnaire was used, respondents might have provided answers not reflective of their actual practices, to appear socially desirable, which may have contributed to reporting bias. In order to minimize the reporting bias, patients were scored for CGI and approved by more than two practicing clinicians to be in right state of mind to answer the questionnaire. In addition, all patients were accompanied by caretakers during the questionnaire sessions and the credibility of the responses were verified by the caretakers and the hospital records. Lastly, the onset of schizophrenia is 20 years old and under. Future studies can be carried out to investigate the relationship between schizophrenia and their risk factors among the younger cohort.

In the present study, several lifestyle and dietary patterns have been found to be significant associated risk factor to SZ severity. On the other hand, the study also highlighted the high prevalence of fungal and parasitic infections among SZ, especially with severe symptoms. Dietary and medical interventions to improve the social deficits and addressing psychiatric symptoms of people with SZ is essential to improve their quality of life.

## Methods

### Sample size estimation

Non-random consecutive sampling was carried out until a target size of 150 was achieved. Sample size estimation was calculated using the formula reported previously^97^_._

Sample size for SZ:

$$n= \frac{\mathrm{Z }{\mathrm{\alpha }}^{2} ({\text{p}})(1-{\text{p}}) }{{E}^{2}}$$

$$n= \frac{{\left(1.64\right)}^{2}(0.004)(1-0.004)}{0.0025}=107$$

where n is the sample size, Z is the statistic corresponding to level of confidence, P is expected prevalence (0.4%), and E is margin error. The level of confidence was set at 90% and the margin error is set at 1%.

### Patient recruitment

Participants between the age of 20 to 70 were enrolled following written informed consent. Participants were evaluated and diagnosed based on the Structured Clinical Interview (SCID) and Diagnostic and Statistical Manual of Mental Disorders, fourth edition^98^, text revision DSMIV-TR (by a certified psychiatrist) and grouped based on Clinical Global Impression of Severity (CGI-S)^99^. In this study, the CGI-S scores range from 1 to 7, where we grouped individuals with mild severity of SZ as 1 and 2 while CGI-S scores of 3 and 4 were used to group individuals with moderate severity of SZ. Patients with severe SZ were grouped under CGI-S score of 5,6 and 7, with 7 being the highest severity. A total of 150 participants with SZ (thrice more than the estimated sample size) were approached from outpatients of University Malaya Medical Centre (UMMC). In addition, sampling was also done among patients visiting National University of Malaysia Medical Centre (NUMMC). SZ patients with a history of drug abuse and have taken anti-parasitic and anti-fungal medications (2 months prior) were excluded from the study. A close-ended questionnaire, comprising questions about standard demographic and lifestyle (dietary habits etc.) was self-administered to participants who consented. For those with poor literacy, caretakers fluent in their respective native languages asked all questions verbally.

In addition, a total of 180 volunteers with similar age groups attending UMMC for various other illnesses were recruited into non-schizophrenic (NS) control group. These volunteers were verified to have no history of schizophrenia. Ethical approval was obtained from the Medical Ethics Committee of the UMMC, Kuala Lumpur, Malaysia (20191226-8107), and the Research Ethics Committee of the National University of Malaysia (UKM PPI/111/8/JEP-2020-725) according to the Declaration of Helsinki.

### Fungal and parasitic screening

In this study presence of fungal and parasites were carried out using a combination of methods. All stool samples were subjected to conventional PCR screening for the detection of (*Blastocystis* sp, *Cryptosporidium* sp., *Micropsoridum, Entamoeba sp., Candida sp. and Aspergillus sp.)*. Simultaneously, xenic culture method (XCD) were used to detect presence of *Blastocystis* sp. while Ziehl Neelsen staining was carried out to detect *Cryptosporidium* sp. *microscopically.* A pea sized (≈250 mg) of stool sample was added into 5 mL Jones medium supplemented with 10% horse serum (Gibco Laboratories, Life Technologies, New York, USA) and incubated at 37 °C^18^. For the next three days, the culture was screened for *Blastocystis* sp. microscopically. Formaline ether sedimentation technique was also used to detect the presence of cysts in the stool. A combination of staining, culture and molecular detection was carried out to increase the sensitivity of the screening. Samples with positive detection for either one test was scored as positive and samples that are negative for all tests are recorded as negative.

## Detection of *Cryptosporidium* sp. using modified Ziehl Neelsen (ZN) staining

Modified ZN staining^100^ was used to determine *Cryptosporidium* sp. Absolute methanol to fix the samples, stain with strong carbol fushin, and acid alcohol to decolorize and counter stain with methylene blue. Then, the sample was viewed under × 40 magnification This technique was carried out by performing fecal smear from the stool or from concentration deposit and let it air dry. After the smear were fixed in methanol for 5 min, proceed with staining using strong carbol fushin for 10 min and rinsed the smear thoroughly with tap water. Next, the smear was decolorized in acid-alcohol for 15 to 20 s and rinsed thoroughly. The smear was counterstained with methylene blue for 30 s and rinsed thoroughly again before letting it air dry before being examined under × 40.

### Intestinal parasite and fungal screening using conventional PCR

Stool samples were obtained from the study participants using a sterile container and stored appropriately within 6 h of collection. Total DNA was extracted from a pea-sized (≈500 mg) stool sample using QIAamp PowerFecal Pro DNA Kits (QIAGEN, USA) according to the manufacturer’s protocol. The extracted DNA template was then used to identify the presence of common intestinal parasites and fungi (*Blastocystis* sp, *Cryptosporidium* sp., *Micropsoridum, Entamoeba sp., Candida sp. and Aspergillus sp.)* using conventional PCR. DNA extracted was quantified and analysed for purity using Nanodrop and agarose gel electrophoresis. A 20-μl reaction volume containing 18 μl of master mix from KAPA (ROCHE), 1 µL of DNA and 0.5µL (10 µM) of forward and reverse primers were prepared. All samples were run along an internal positive control which was used as a comparison for the amplicon size. The list of primers and conditions of the PCR protocol is listed in Supplementary Table S1.

### Statistical analysis

Collected data were cleaned using Statistical Package for Social Science (SPSS) version 23.0 software (IBM Corporation, Armonk, NY, USA). Incomplete questionnaires were filtered out and only complete questionnaires were used for analysis. Continuous data were reported as median and interquartile range (IQR, 25th percentile to 75th percentile) due to non-normal distribution assessed via Shapiro–Wilk test. Count data were expressed as frequency (n) and percentage (%). Univariable and multivariable binary logistic regression analyses were used to identify the risk factors associated with SZ. In univariable logistic regression, variables that were found associated with SZ at *p* < 0.25 based on Wald statistics were included in the subsequent multivariable logistic regression^101^. Backward stepwise elimination was conducted to remove less important variable and maintain only a final model with statistically significant (*p* < 0.05) variables. Ordinal logistic regression was performed to identify the association between studied variables and CGI-S score level using Stata version 15 software (StataCorp, College Station, TX, USA). Brant test was used to assess the proportional odds assumption of the model, and variables that violate the assumption were excluded from the model via backward stepwise elimination^101^. Only statistically significant (*p* < 0.05) variables were included in the final ordinal logistic regression model. To evaluate the goodness of fit of regression models, Hosmer–Lemeshow test was applied for binary logistic regression model whereas the ordinal version of this test was applied for ordinal logistic regression models. UpSet diagrams were plotted using ComplexHeatmap package in R version 4.2.2 (R Foundation for Statistical Computing, Vienna, Austria).

### Ethics approval and consent to participate

Ethical approval for administration of questionnaire and the collection of stool samples was obtained from the Medical Ethics Committee of University Malaya Medical Centre (UMMC), Kuala Lumpur, Malaysia (20191226–8107) and Research Ethics Committee of the National University of Malaysia (UKM PPI/111/8/JEP-2020–725) according to the Declaration of Helsinki. A written consent was obtained from all the participants or nearest kin prior to sample collection.

### Supplementary Information

Supplementary Information 1.

Supplementary Information 2.

Supplementary Information 3.

## Data Availability

The authors confirm that the data supporting the findings of this study are available within the article. Raw data that support the findings of this study are available from the corresponding author, upon reasonable request. Correspondence and requests for materials should be addressed to AR.
