# Supplementary material for: Establishing associated risk factors, including fungal and parasitic infections among Malaysians living with schizophrenia
Source: Sci Rep. 2024 Jan 3;14:385. doi: 10.1038/s41598-023-50299-7 (PMC10764362; doi:10.1038/s41598-023-50299-7)
Supplement: Supplementary file 1 — Supplementary Information 1. [file 41598_2023_50299_MOESM1_ESM.docx]

**Supplementary table**

**Supplementary Table S1**

List of Primers used for the screening of common fungal and parasitic infections from stool samples.

**General PCR Protocol.**

A pea sized (≈500mg) stool sample was subjected to DNA extraction using QIAamp Power Fecal Pro DNA kit (QIAGEN) according to the manufacturer’s instruction. DNA extracted was quantified and analysed for purity using Nanodrop and agarose gel electrophoresis. A 20-μl reaction volume containing 18 μl of master mix from KAPA(ROCHE), 1µL of DNA and 0.5µL(10µM) of forward and reverse primers were prepared. All samples were run along a positive control.

| Organism | Sequences | T_m_(˚C) | Amplicon Size(bp) | Reference |
| --- | --- | --- | --- | --- |
| Microsporidium (*E*.bieneusi), | F: GAAACTTGTCCACTCCTTACG  R: CCATGCACCACTCCTGCCATT | 55 | 607 | ^1^ |
| *Blastocystis* sp. | F: AGTAGTCATACGCTCGTCTCAAA  R: TCTTCGTTACCCGTTACTGC | 68 | 320-342 | ^2^ |
| *Cryptosporidium* sp. | F: TAAACGGTAGGGTATTGGCCT  R: CAGACTTGCCCTCCAATTGATA | 55 | 240 | ^3^ |
| *Entamoeba* sp. | **Primary PCR**    F: TAA GAT GCA GAG CGA AA  R: GTA CAA AGG GCA GGG ACG TA  **Secondary PCR**  F: AAG CAT TGT TTC TAG ATC TGA G  R: AAG AGG TCT AAC CGA AAT TAG | 48  48 | 174-553* | ^4^ |
| *Candida* sp. | F: TTTATCAACTTGTCACACCAGA  R: ATCCCGCCTTACCACTACCG | 50 | 272 | ^5^ |
| *Aspergillus* sp. | F: CAGCGAGTACATCACCTTGG  R: CCATTGTTGAAAGTTTTAACTGATT | 55 | 521 | ^6^ |

**E. histolytica*: 439bp, *E. dispar* : 174bp, *E. moshkovskii* : 553bp

1 Hassan, N.-A. *et al.* Molecular diagnosis of microsporidia among immunocompromised patients in Kuala Lumpur, Malaysia. **99**, 1562 (2018).

2 Poirier, P. *et al.* Development and evaluation of a real-time PCR assay for detection and quantification of Blastocystis parasites in human stool samples: prospective study of patients with hematological malignancies. **49**, 975-983 (2011).

3 Bairami, A., Rezaei, S. & Rezaeian, M. J. I. J. o. P. Synchronous identification of Entamoeba histolytica, Giardia intestinalis, and Cryptosporidium spp. in stool samples using a multiplex PCR assay. **13**, 24 (2018).

4 Lau, Y. L. *et al.* Real-time PCR assay in differentiating Entamoeba histolytica, Entamoeba dispar, and Entamoeba moshkovskii infections in Orang Asli settlements in Malaysia. **6**, 1-8 (2013).

5 Taira, C. L. *et al.* A multiplex nested PCR for the detection and identification of Candida species in blood samples of critically ill paediatric patients. **14**, 1-7 (2014).

6 Mohd Zainudin, N. A. I., Abd Murad, N. B., Aris, A. & Hussain, N. H. J. P. D. First report of Aspergillus niger causing fruit rot of bilimbi in Malaysia.
