# Supplementary material for: Establishing associated risk factors, including fungal and parasitic infections among Malaysians living with schizophrenia
Source: Sci Rep. 2024 Jan 3;14:385. doi: 10.1038/s41598-023-50299-7 (PMC10764362; doi:10.1038/s41598-023-50299-7)
Supplement: Supplementary file 2 — Supplementary Information 2. [file 41598_2023_50299_MOESM2_ESM.docx]

**Supplementary Table S2**

Supplementary Table S2 shows the total fungal and parasite detected via microscopy and molecular method among NS cohort group.

| Column1 | Total | Micros Microscopy copy | Molecular Column3 |
| --- | --- | --- | --- |
| *Blastocystis* sp | 9 | 9 | 9 |
| *Entamoeba* sp. | 0 | 0 | 0 |
| Microsporidium | 15 | 5 | 15 |
| *Cryptosporidium* | 8 | 3 | 6 |
| *Candida* sp. | 15 | - | 15 |
| *Aspergillus* sp. | 5 | - | 5 |
| *T*otal | 52 | 17 | 50 |
